# Exercise improves aging-related decreased angiogenesis through modulating VEGF-A, TSP-1 and p-NF-Ƙb protein levels in myocardiocytes

**DOI:** 10.34172/jcvtr.2020.21

**Published:** 2020-05-18

**Authors:** Bagher Pourheydar, Abdolrahman Biabanghard, Reza Azari, Naser Khalaji, Leila Chodari

**Affiliations:** ^1^Department of Anatomical Sciences, School of Medicine, Urmia University of Medical Sciences, Urmia, Iran; ^2^Neurophysiology Research Center, Cellular and Molecular Medicine Institute,Urmia University of Medical Sciences, Urmia, Iran; ^3^Department of Physiology, Faculty of Medicine, Urmia University of Medical Sciences, Urmia, Iran; ^4^Faculty of Medicine, Urmia University of Medical Sciences, Urmia, Iran

**Keywords:** Aging, Heart, Angiogenesis, Exercise

## Abstract

***Introduction:*** Aging-dependent decline in the angiogenesis of heart is a risk factor for cardiovascular disease. This study was aimed to characterize effect of exercise on angiogenesis alterations and molecular mediators which are related to angiogenesis in the heart under aging condition.

***Methods:*** Twenty-one male Wistar rats were assigned into three groups: young, aged, and exercise. Aged animals in the exercise group run on treadmill for 8 weeks. At the end, heart samples were collected and used for histological evaluation , determination of angiogenesis by immunostaining for PECAM-1/ CD31 and expressions of vascular endothelial growth factor-A (VEGF-A), thrombospondin-1 (TSP-1) and nuclear factor kappa B (NF-κB) levels by ELISA. P<0.05 is considered as statistically significant.

***Results:*** Our results showed that angiogenesis, and VEGF-A levels were significantly decreased, TSP1 (*P* >0.0001) and p-NF-κB (*P* >0.001) levels were significantly increased in the heart of aged group compared to young group. Exercise group showed significant increase in angiogenesis, VEGF-A (*P* >0.0001), and p-NF-κB (*P* >0.001) and showed significant decrease in TSP-1 levels (*P* >0.001) compared to aged group. Moreover, compared to the young group, aged group showed histological changes in the heart, such as interstitial edema, and congestion, whereas, treatment with exercise improved these undesirable changes in the heart of exercise groups.

***Conclusion:*** These findings indicated that aging-related decrease in angiogenesis in the heart may mediated by downexpression of VEGF-A and overexpression of TSP-1 proteins. Also, we showed that p-NF-κB protein was increased in the heart of aged rats, this probably mediated by compensatory mechanism. It was also showed that exercise as novel non-pharmacological therapy modifies VEGF-A and TSP-1 and increases p-NF-κB protein levels in the aged heart.

## Introduction

Aging is the consequence of the gradual accumulation of many alterations in the body that accompanied with decline of the efficiencies of normal physiological functions and capacity to maintain homeostasis.^1^ The fast growth of elderly population in the world is awareness for age-related diseases, including interest in the study of the aging human heart.^2^ Aging is associated with a dominant risk factor for most forms of cardiovascular diseases, including heart attacks and decreased ischemic tolerance that leads to increase in mortality rate in the elderly population.^3^

Reduced neovascularization and endothelial malfunction probably contribute to the age-related cardiovascular diseases.^4^ Neovascularization, including angiogenesis and vasculogenesis, shows a vital impact on the delivery of oxygen, nutrients, and other mediators at the injury and ischemic sites.^5^ Angiogenesis is controlled by the balance between several pro and anti-angiogenic agents activity. There are more factors and cytokines that involved in the angiogenesis process such as vascular endothelial growth factor-A (VEGF-A), thrombospondin-1 (TSP-1) and nuclear factorkappa B (NF-κB).^6^

VEGF-A is most likely primary factor involved as both a diffusible attractant for migrating endothelial cells and substrate-bound vessel stabilization factor once vascular tubes are formed.^7^ It was demonstrated that there is a trend for decreased expression of VEGF-A in the heart of aged male rats.^8^ Conversely, TSP-1 is a strong inhibitor of angiogenesis acting as modulators of the angiogenic response. Previous study have shown that TSP-1 inhibits both basic fibroblast growth factor– and VEGF-mediated endothelial cell proliferations.^9^ TSP-1 is expressed at very low levels in normal adult mammalian tissues but is markedly upregulated in aged tissue.^10^ NF-κB is another important molecule in the process of angiogenesis that has been recently found to regulate endothelial cell integrity and vascular homeostasis.^11^ Although, much is known about angiogenesis in general, age-related decreased in angiogenesis is not well defined. Also, most studies of angiogenesis in aging have been achieved by the use of varied pathological models such as ischemia, glomerulonecrosis, and excisional wounds in the rat, rather than on healthy old animals.^8,12,13^

In this study we desired to characterize the changed mechanisms (VEGF-A, TSP-1 and NF-κB) that influence decreased angiogenesis in healthy aged rat relative to young rat. Whereas, induction of angiogenesis is a promising therapeutic approach for ischemic diseases but there are limited medical therapies to improve angiogenesis in aging. So providing guidelines for reducing age-associated cardiac diseases seem to be necessary. Recently, the positive effects of physical activity in reduce of pathogenesis of cardiomyopathy have been shown.^14^ However, the pro-angiogenesis corresponding molecular mechanisms used by exercise in the aged heart are unknown. So, in the present study, two aims have been pursued, the first aim was to investigate the effect of aging on VEGF-A, TSP-1, and p-NF-κB (phosphorylated and activated forms of NF-κB) protein levels alteration in the cardiac tissue and the second aim was determination of the possible molecular mechanism (VEGF-A, TSP-1 and p-NF-κB) that employed by exercise to improve the elderly-related changes in the heart.

## Materials and Methods

### Animals and study design

Animals were housed at a constant room temperature (24°C), a relative humidity for 50%, and a 12 h dark/light cycles that possession food and water ad libitum. All rats were kept at four rats/cage during of study. We have taken all steps to minimise the animals’ pain and suffering. IP injection of ketamine and xylzine were used to anesthetize the animals at the end of the study.

To calculate the sample size, we used the mean difference formula between the two groups with 95% confidence and 90% power and using the results of da Silva et al.^15^ The sample size by using the formula below equals six (n = 6) and with a 20% probability of loss, the sample size was assumed 7 rats for each group (n = 7).

$$n=\frac{{\left(Z_{1-\frac{\alpha }{2}}+Z_{1-\beta }\right)}^{2}(\delta_{1}^{2}+\delta_{2}^{2})}{{\left(\mu_{1}-\mu_{2}\right)}^{2}}$$

Accordingly, seven young (3 months, 200–250 g) and fourteen elderly (26 months, 400–450 g) same male Wistar rats were randomly divided into three groups regardless of the specific feature, (n = 7):

1. Sedentary young group (con): young rats that were kept without any intervention for 2 months.
2. Sedentary aged group (aged): aged rats that were kept without any intervention for 2 months.
3. Exercise group (aged+ exe): aged rats that performed exercise for 2 months.

### Exercise protocol

At first, the aged rats in the exercise group were adapted to treadmill running over a one-week period. Animals in the exercise group ran on a treadmill according to training program, as a mild exercise consists of running at a speed of 17 m/min, without inclination, for 10 minutes on the first day with progressive increase every day (+5 minutes), reaching 30 minutes on the 5th day, which was maintained for the next seven weeks. Sedentary rats were handled similar to exercised rats, without training.^16^ All experiments were carried out at 9-12 am.

### Tissue processing and protein measurement

On the final day of experiment, all animals were anesthetized with ketamine (80 mg/kg) and xylazine (5 mg/kg); heart tissues were immediately excised, washed with saline 0.9% and frozen immediately with liquid nitrogen. Tissue samples from the left ventricle were used for measurement of protein levels and were stored in −70°C temperatures until evaluation of VEGF-A, TSP-1, and NF-κB p65 (phosphorylated and activated form) levels. Then, the samples were homogenized in PBS buffer (pH 7.2 to 7.4) and centrifuged for 20 minutes at 1000 g and at 4°C. Resulting supernatants were removed, and target proteins were extracted. VEGF-A, TSP-1, and p-NF-κB levels were measured by high-sensitivity ELISA kits (Zelbio, Germany) according to the manufacturer’s instructions.

### Immunostaining for PECAM-1/CD31

For the investigation of angiogenesis in the cardiac tissue, samples from left ventricle were immersed into 10% formalin after excision, embedded in paraffin, and cut into 4 μm-thick slices. Sections were deparafinized in xylene and dehydrated in a graded series of ethanol. Slides were incubated sequentially in proteinase K and treated by 0.3% hydrogen peroxide to block endogenous peroxidase activity. Sections were overlaid by primary antibody CD31 (Santa Cruz, USA) a marker of angiogenesis and incubated at 4°C overnight. Sections were then washed and incubated with standard avidin–biotin complex (ABC; Santa Cruz) according to the manufacturer’s instructions.

### Assessment of immunostaining

Depending on the size of the sample section, 3 to 5 (1 mm^2^) areas were selected randomly by light microscope (Olympus BX 40, Japan) and were evaluated at the magnification ×40. Intensity scoring for each positive endothelial cell cluster of immunoreactivity within the selected field obtained by ×200 magnification. For assessment of immunostaining, the granulated tissue was used as a positive control, and the intensity of the staining was scored as 0 (<10%); 1 (10% to 25%); 2 (25% to 50%); 3 (50% to 75%) or 4 (75% to 100%).^18^

### Histological evaluation

Sections of 5 μm were taken from left ventricle parafinized samples, stained with hematoxylin & eosin (H&E), and were examined morphologically under light microscope (Olympus BH-2, Tokyo, Japan) in a blinded manner.

### Statistical analysis

Statistical analysis was performed using SPSS 16 (SPSS/PC-16, SPSS Inc., USA). Data distribution were controlled using the Kolmogorov–Smirnov test. The data were normally distributed and analyzed using parametric techniques. The statistical differences between the groups were tested by conducting a one-way ANOVA, and then the Turkey post hoc test. The data obtained from each test are presented as the mean ± standard deviation (SD), and *P* < 0.05 is considered as statistically significant.

## Results

### Effects of exercise training on VEGF-A protein level in the heart tissue

**Figure 1:**
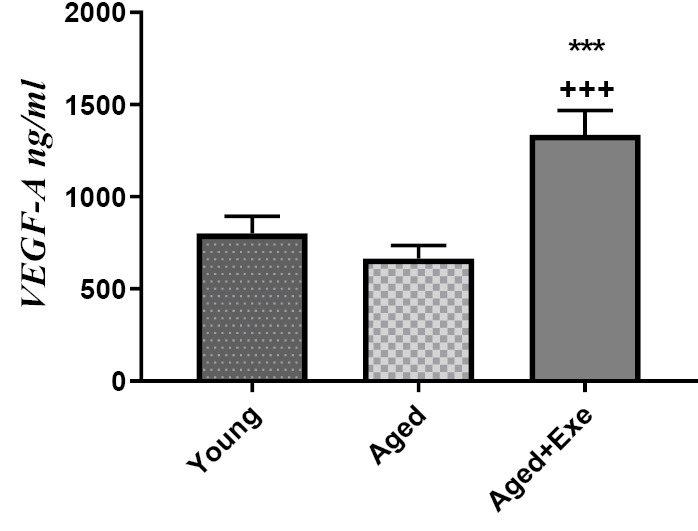
Effect of exercise treatment for 8 weeks on VEGF-A levels in cardiac tissue. Bars represent the mean±SD (n=7), *** *P*  < 0.001 vs. the Young group, ^+++^*P*  < 0.001 vs. the Aged group.

The results obtained by ELISA assay showed that VEGF-A protein level in the heart tissue of the aged group had no significant change compared to the control group. As, 8 weeks treatment with exercise significantly (*P* <0.001) increased VEGF-A protein levels in heart tissue of aged rats compared to sedentary aged group. It was very interesting that heart tissue in the aged -exercise group had a significant (*P* <0.001) increased level of VEGF-A compared to the young group (Figure 1).

### Effects of exercise training on TSP-1 protein level in heart tissue

**Figure 2:**
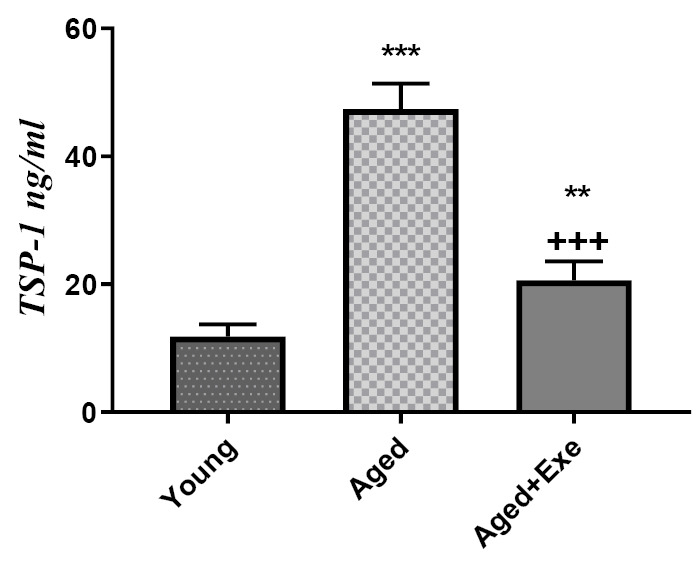
Effect of exercise treatment for 8 weeks on TSP-1 levels in cardiac tissue. Bars represent the mean ± SD (n=7), **P  < 0.01, ***P  < 0.001 vs. the young group, ^+++^*P*  < 0.001 vs. the Aged group.

Figure 2 shows that TSP-1 protein levels of heart tissue in the aged group is significantly (*P*  < 0.001) higher compared to the young group. Also, eight weeks exercise treatment decreased TSP-1 protein levels significantly (*P*  < 0.001) compared to the aged sedentary group but it was still significantly higher than that of the young group (*P*  < 0.01).

### Effects of exercise training on p-NF-κB protein level in the heart tissue

**Figure 3:**
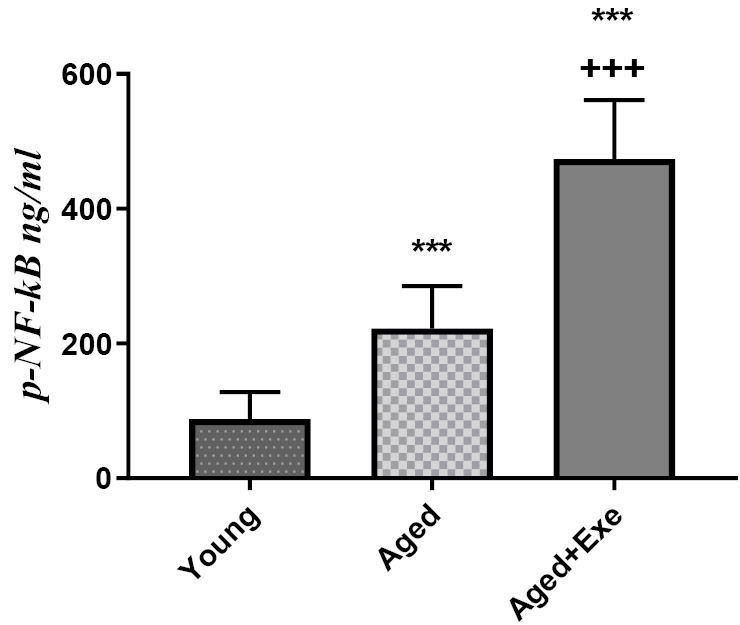
Effect of exercise treatment for 8 weeks on p-NF-kb levels in cardiac tissue. Bars represent the mean ± SD (n=7), ***P  < 0.001, ***P  < 0.001 vs. the young group, ^+++^*P*  < 0.001 vs. the aged group.

According to Figure 3, elderly rats in the aged group significantly (*P*  < 0.001) have an increasing level of p-NF-κB protein in the heart tissue compared to the young group. Eight weeks exercise training significantly increased the p-NF-κB protein level in the heart tissue comparing to the sedentary aged group (*P*  < 0.001) and comparing to the young group (*P*  < 0. 001).

### Effects of exercise training on angiogenesis in the heart tissue

**Figure 4:**
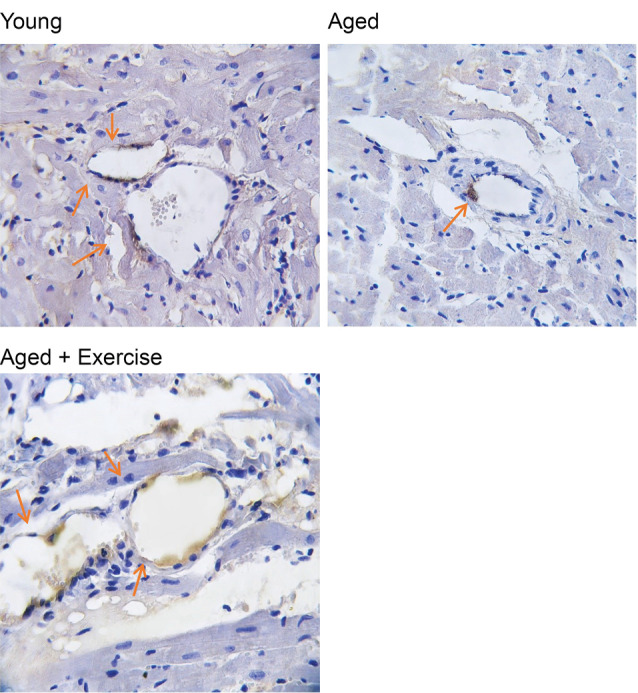
Immunohistochemical detection of CD31 in heart tissue. Brown stained tissues show CD-31 immunostained endothelial cells in: (A) Control; (B) Aged; and (C) Aged + exercise. The number and intensity of immunostaining for CD31 (Red arrow) decreased in the aged group compared to the young group. Exercise treatment increased angiogenesis in Aged+ Exercise group compared to the Aged group (Magnification was x400).

**Figure 5:**
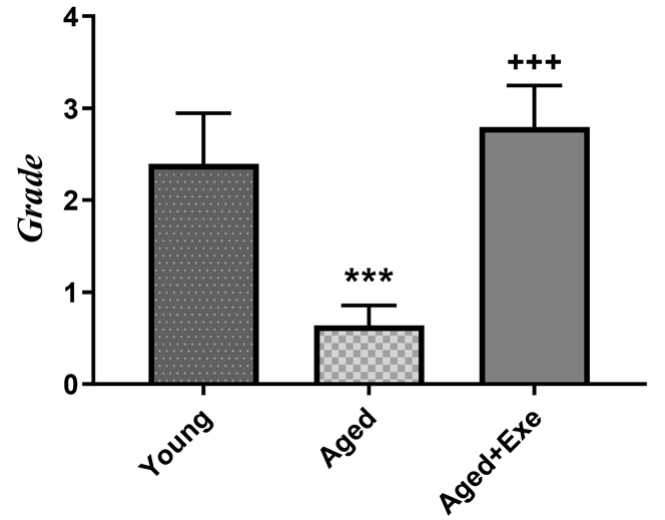
Effect of exercise treatment for 8 weeks on angiogenesis levels in cardiac tissue. The intensity of the staining was scored as: 0 (<10%); 1 (10-25%); 2 (25-50%); 3 (50-75%); and 4 (75-100%). Bars represent the mean ± SD (n=7), ***P  < 0.001, vs. the Young group, ^+++^*P*  < 0.0001 vs. the Aged group.

Immunostaining with CD31 marker was done for the assessment of angiogenesis in the heart. Brown stained tissues show CD-31 immunostained endothelial cells. For assessment of immunostaining, the granulation tissue was used as a positive control, and the intensity of the staining was scored as 0 (<10%); 1 (10% to 25%); 2 (25% to 50%); 3 (50% to 75%) or 4 (75% to 100%). According to figures 4 and 5, statistical analysis of our immunohistochemical study showed that angiogenesis was decreased significantly (*P*  < 0.001) in the aged group compared to the young group. Also, eight weeks of exercise treatment in the aged group significantly (*P*  < 0.001) increased angiogenesis in the heart compared to the aged group (Figures 4 and 5) and the level of angiogenesis in the exercise group was found to be similar to that in the young animals.

### Effects of exercise training on myocardial histological changes

**Figure 6:**
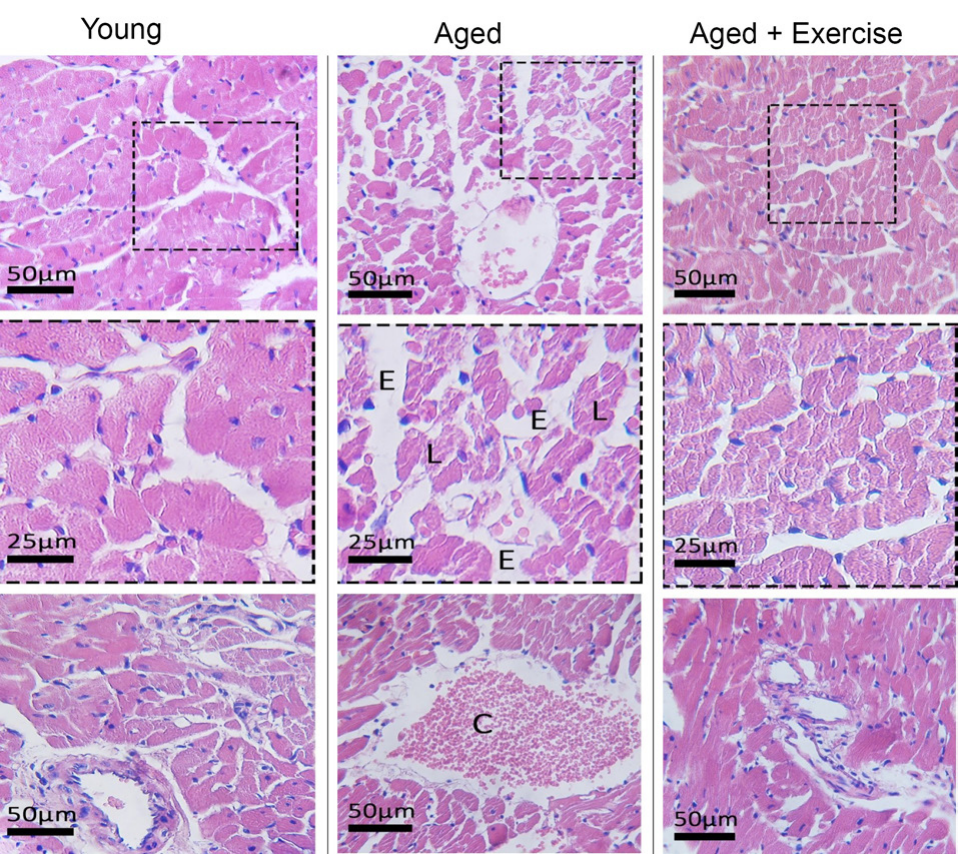
The histological light microscopy images of hematoxylin and eosin stained sections of heart tissue from each group are shown (×40 HE). There were interstitial edema (E), cell lysis (L), and congestion (C) in the aged group. Improved tissue structure was seen in the aged+ exercise group that done exercise for 8 weeks.

Hematoxylin and eosin staining was done to assess the effect of exercise treatment on the heart tissue alterations in the aged animals (Figure 6). According to Figure 4, aged group had an increased interstitial edema, and congestionin comparison to the young group.Furthermore, eight weeks exercise in the aged + exercise group showed a decreasing effect on myocardial tissues damage in comparison to untrained aged animals. Also, animals in exercise group had a marked attenuation of interstitialedema, and congestion as found in the control young group.

## Discussion

Several studies have shown that aging induces gradual structural and functional injuries to the heart that somewhat associated with decreased angiogenesis in the cardiac tissue.^19,20^ So far, the exact pathological molecular mechanisms of decreased angiogenesis are remained unclear and there is no remarkable and effective treatment for age-related heart diseases. So, in this study, we investigated the effects of aging and exercise training on the histological changes, expression of angiogenesis related proteins and capillarization in the heart tissue of rats. Our findings showed that aging impaired expression of VEGF- A, TSP-1, and NF-κB in the heart tissue and induced undesirable histological changes such as tissue organization and decreased vascularization in the heart tissue. Furthermore, we also demonstrated that exercise as non-pharmaceutical treatment reduced adverse effects of aging by ameliorate expression of VEGF- A, TSP-1, and NF-Kb in the heart tissue of aged rats and decreased histopathological damage in the aged myocardium.

Considering the previous facts, obviously, it is expected that further deterioration of age-related cardiac diseases were associated with the reduction of total capillary density and an alteration in the local balance among angiogenesis association competing cytokines and growth factors in the heart.^20^ Among dozens of molecular mediators related to angiogenesis pathways, mediators such as VEGF-A, TSP-1, and NF-κB play important roles in the regulation of new vessel formation.^21,22^ VEGF-A is a powerful mitogen of endothelial cells and a main stimulating factor in angiogenesis which has been studied widely in angiogenesis research.^7^ In the present study, we showed that capillary density and VEGF-A protein levels in the cardiac tissue were dramatically decreased in the aged rats. The results of the present study are in line with the results of a previous study demonstrating that there was a trend for decreased expression of VEGF-A in the heart of aged animal models.^23^

Another novel finding in this study was the increased expression of TSP-1 in the heart tissue of the aged rats. It is proven that thrombospondin does not function as structural proteins but acts as modulators of the angiogenic response.^22^ The regulation of angiogenesis by TSP-1 and TSP-2 has been widely studied; it has been shown that the TSP family suppresses the bioavailability and activity of VEGF, induces apoptosis in the endothelial cell, inhibits migration of the endothelial cell, and suppresses nitric oxide signaling.^22^ In line with our work, concurrent study in aged animals have confirmed that TSP-2 is up regulated in the aged tissue.^13^ The cause of accentuated TSP-1 synthesis in aging condition is vague; however, it is clear that oxidative stress has been elevated in old age and on other hand Chen and et al in a study on primary rat astrocytes , suggests that TSP1 levels has been elevated in astrocytes in response to oxidative stress.^24^ It seems that aging increases TSP1 levels which act as chief negative regulators of neovascularization through increasing oxidative stress levels. In age-related heart failure, expression of cluster 17–92 miRNA members was down-regulated. Accordingly, TSP1 was increased. Importantly, these expression changes occurred only in cardiomyocytes, not in fibroblasts.^25^

Recently, it is widely accepted that transcription factor NF-κB plays various roles in associated with several features of angiogenesis.^21^ NF-κB signaling has been found to adjust endothelial cell integrity and vascular homeostasis in vivo.^26^ In addition, several studies report that angiogenesis inhibition is associated to NF-κB activation.^27^ Many suggestions have been put forward to clarify this dual action. The emerging general assumption is that the ending outcome of NF-κB activation depends on cell type, stimulus, and context of activation.^28^ Our data illuminated that the aged rats had dramatically high levels of p-NF-κB compared to the young rats. It is interesting that the promoters of TSP-1 and -2 contained NF- Ƙb binding sites, in agreement with our results, it can be assumed that increased level of NF-κB in aging condition is one cause for TSP-1 increased levels in aged rat. Considering the mentioned issues, decreased proangiogenic factors in the aged cardiac tissue can play a main role in age related cardiac diseases. Consequently, excessive consideration has been focused on the application of angiogenesis augmentative agents. Furthermore, it has been proven that regular exercise has multi-system anti-aging effects and there is no doubt about its improving effect on angiogenesis.^23^ However, not obvious research has been conducted so far on the effects of exercise on angiogenesis and its subsequent signaling pathway in old age. Thus, this study aimed at precisely filling this loophole. The results showed that levels of VEGF-A, p-NF-κB and capillary density were increased and TSP-1 was decreased in exercise-treated- aged rats compared with sedentary- young rats.

In agreement with the study,Iemitsu et al, have shown that exercise training during old age improves malfunction of heart, and the underlying mechanism is up regulation of VEGF angiogenic signaling cascade in the aged heart.^8^ Overall, histopathological comparison of the cardiac tissue between the aged rats and the aged treated group showed that an observable angiogenic reaction occurred as a result of exercise.^23^ Moreover, the results showed that the levels of p-NF-κB in the cardiac tissue was increased in aged rats compared to young rats and interestingly, the increased levels of p-NF-κB in aged rats were multiplied by exercise training. NF-κB has been newly associated with numerous features of angiogenesis.

As mentioned, several studies report that angiogenesis inhibition is associated to NF-κB activation.^29^ In contrast to the inhibition effects of NF-κB on angiogenesis, recent reports suggest that in certain situations NF-κB can promote angiogenesis.^30^ In our unpublished research, we showed that NF-κB levels were increased in diabetes and exercise reversed this effect and promoted angiogenesis in sciatic nerve of diabetic rats in exercise group. Also, Liu et al demonstrated that treadmill exercise training have a decreasing effect on NF-κB expression in muscle of diabetic mice.^31^

In this study, we showed that exercise increased NF-κB levels in the cardiac tissue. Additionally, high level of new vessel formation obtained by the immunostaining test in the aged trained group displayed an enhanced angiogenesis attribute for exercise in aging condition. Gordon et al expressedthat cardioprotective role of NF-κB signaling has been evident for a number of years.^32^ Some studies have showed a potent role for the NF-κB in the regulation of cardiac myocyte survival through repression of TNF-induced apoptosis.^33^ It seems that outcomes of NF-κB signalingwill depend on additional criteria, such as the timing and duration of signaling, as well as other environmental cues and cellular context.^32^

So, we posit a novel role for NF-κB that this molecule plays a proangiogenesis role in aged cardiac tissue. Also, most studies of angiogenesis in aging have been confounded by the use of varied pathological models such as ischemia, glomerulonecrosis, and excisional wounds in the rat models, rather than on healthy old animals. Also, according to the obtained data, it can be assumed that the increased NF-κB levels in the heart of aged rats can depict a protective effect. However, further research is needed to clarify the function of cardiac angiogenesis related molecules in aging condition.

### Limitations of the study

It is better that immunohistochemical techniques for NF-κB were performed parallel immunohistochemical techniques for CD31, for confirmation of NF-κB pro-angiogenesis role.

## Conclusion

In conclusion, our data demonstrated the novel finding that angiogenesis in the heart of healthy aged rats were diminished as a result of impaired production of VEGF-A as a proangiogenesis agent and TSP-1 as an antiangiogenesis agent. Additionally, we showed that p-NF-κB levels as an angiogenesis regulator, were increased in the aged rats, probably throughout a compensatory mechanism. The present study also revealed that exercise training reversed the aging-induced decrease in VEGF-A and aging-induced increase in TSP-1 levels. Also, exercise training increased NF-κB levels higher than that in the sedentary aged rats. Accordingly, we assume that exercisecan be a novel non- pharmacological therapy window for age-related cardiac diseases.

## Competing interests

The authors declare that they have no conflict of interest.

## Ethical approval

The study was approved by the Animal Ethics Committee of the Urmia University of Medical Sciences, (ethical code: IR.UMSU.REC.1397.149).

## Acknowledgments

Financial support for this investigation by the Faculty of Medicine, Urmia University of Medical Sciences through grant (Grant No. IR.UMSU.REC.1397.149), is gratefully acknowledged.
